# The emerging roles of circular RNA-mediated autophagy in tumorigenesis and cancer progression

**DOI:** 10.1038/s41420-022-01172-5

**Published:** 2022-09-14

**Authors:** Yuan Yuan, Xiaojing Zhang, Xinmin Fan, Yin Peng, Zhe Jin

**Affiliations:** Guangdong Provincial Key Laboratory of Genome Stability and Disease Prevention and Regional Immunity and Diseases, Department of Pathology, Shenzhen University Health Science Center, 518060 Shenzhen, Guangdong People’s Republic of China

**Keywords:** Tumour biomarkers, Oncogenes

## Abstract

Circular RNA (circRNA) is characterized by a specific covalently closed ring structure. The back-splicing of precursor mRNA is the main way of circRNA generation, and various cis/trans-acting elements are involved in regulating the process. circRNAs exhibit multiple biological functions, including serving as sponges of microRNAs, interacting with proteins to regulate their stabilities and abilities, and acting as templates for protein translation. Autophagy participates in many physiological and pathological processes, especially it plays a vital role in tumorigenesis and carcinoma progression. Increasing numbers of evidences have revealed that circRNAs are implicated in regulating autophagy during tumor development. Until now, the roles of autophagy-associated circRNAs in carcinoma progression and their molecular mechanisms remain unclear. Here, the emerging regulatory roles and mechanisms of circRNAs in autophagy were summarized. Furtherly, the effects of autophagy-associated circRNAs on cancer development were described. We also prospected the potential of autophagy-associated circRNAs as novel therapeutic targets of tumors and as biomarkers for cancer diagnosis and prognosis.

## Facts

- circRNAs are implicated in diverse physiological processes, including autophagy. They are also associated with the pathogenesis of numerous diseases, such as cardiovascular diseases, neurological disorders, and cancer.
- circRNAs have both stimulatory and inhibitory effects on autophagy.
- circRNA-mediated autophagy plays vital roles in multiple aspects of tumor progression, especially the development of chemoresistance in cancer.
- The expression patterns of circRNAs are associated with the clinicopathological characteristics of cancer.
- circRNAs are considered to represent novel therapeutic targets and potentially useful diagnostic and prognostic biomarkers of cancer.

## Open questions

- How do circRNAs regulate autophagy in the context of cancer?
- How does circRNA-mediated autophagy affect tumorigenesis and cancer development?
- Should we promote or inhibit circRNA-mediated autophagy to suppress cancer progression?
- Whether targeting autophagy-associated circRNAs can be novel strategies for cancer treatment?

## Introduction

Circular RNA (circRNA) is a class of endogenous RNA molecules with special covalent closed-loop structure. CircRNAs are mainly created from precursor mRNA (pre-mRNA) back-splicing, and they have been identified in a variety of eukaryotes and viruses [1, 2], and exhibit developmental-stage-specific and tissue-specific expression patterns [3, 4]. When they were first discovered nearly 50 years ago, these RNA molecules received little attention and were thought to be derived from errors in the splicing of mRNA and thus lacking in biological importance. However, recently researchers have found that circRNAs are implicated in diverse physiological processes, including autophagy [5] and immunity [6, 7]. They are also associated with the pathogenesis of numerous diseases, such as cardiovascular diseases [8], neurological disorders [9], chronic inflammatory diseases [10, 11], and cancer [12, 13]. The roles played by circRNAs in tumorigenesis and cancer progression have also recently been revealed. Numerous dysregulated circRNAs have been identified in diverse carcinomas, such as colorectal cancer (CRC) [14, 15], gastric cancer (GC) [16–18], basal cell carcinoma [19, 20], and hepatocellular carcinoma (HCC) [21, 22], and their expression was related to the clinicopathological features of cancer patients. We previously analyzed the expression profiles of circRNAs in five pairing GC and corresponding adjacent tissues, using circRNA sequencing with linear RNA depletion [16]. We totally identified 45,783 circRNAs from these samples, including 79% exonic, 1% intronic, 4% intergenic, 15% sense overlapping, and 1% antisense[16]. These circRNAs may participate in GC development. It has become apparent that circRNAs play vital roles in cell death [23], chemoresistance [24], metastasis [25], the maintenance of cancer-initiating cells [26], immune evasion [27], and angiogenesis [28] in cancer. circRNAs usually show higher stability compared with that of their linear host gene, presumably due to their cyclic structures, which render them resistant to exonuclease-mediated degradation. Furthermore, some circRNAs are detectable in body fluids, including saliva, urine and peripheral blood, which suggests the possibility of their being used as non-invasive biomarkers [29]. Thus, circRNAs show great potential as diagnostic and prognostic biomarkers and as therapeutic targets for cancer therapy.

Autophagy, as a highly conserved catabolic process, plays key roles in maintaining a balance between cellular survival and death, which regulates the degradation and recycling of intracellular materials, including damaged organelles and misfolded proteins. It renders cells resistant to survival stress (such as nutritional deficiency and hypoxia), via providing materials and energy for synthesis of new cellular components and thereby restoring cellular homeostasis [30]. Three types of autophagy (macroautophagy, microautophagy, and chaperone-mediated autophagy) have been identified [31]. This review is focused on macroautophagy, characterized by the formation of autophagosomes that possess a double membrane structure. In macroautophagy (hereinafter referred to as autophagy), after being enveloped by autophagosomes, damaged cellular components are transported to lysosomes for degradation and recycling. To date, the role autophagy plays in cancer development remains controversial. On one hand, autophagy suppresses tumorigenesis by maintaining genome stability and homeostasis of cellular metabolism. On the other hand, it participates in reprogramming cellular microenvironment following the establishment of cancer and protects cancer cells from diverse survival stresses [32]. Autophagy also helps cancer cells escape from anti-tumor immune responses mediated by natural killer cells and cytotoxic T-lymphocytes[33]. Furthermore, increasing evidence has revealed the double role circRNAs play in autophagy regulation. Some circRNAs have been found to promote autophagy. A study by Yang and colleagues determined that circRHOBTB3 functions as a microRNA (miRNA) sponge, and facilitates autophagy via circRHOBTB3/miR-600/NACC1 axis, leading to increased cellular proliferation in pancreatic ductal adenocarcinoma (PDAC) [34]. Conversely, other circRNAs negatively regulate autophagy. circUBE2Q2 was shown to suppress STAT3-mediated autophagy via sponging miR-370-3p but promote tumorigenicity in GC [35]. Thus, to develop novel circRNA-based therapeutic strategies for cancer, the mechanisms of how circRNAs regulate autophagy and how circRNA-regulated autophagy affects tumorigenesis and progression must be urgently clarified.

Here, the emerging findings in relation to the regulatory roles circRNAs play in autophagy are summarized and these molecules’ effects on cancer development and progression are described. Furtherly, the potential of circRNAs as novel biomarkers of cancer diagnosis and prognosis and therapeutic targets for tumor treatment is also prospected.

## circular RNA

### circular RNA biogenesis

**Fig. 1:**
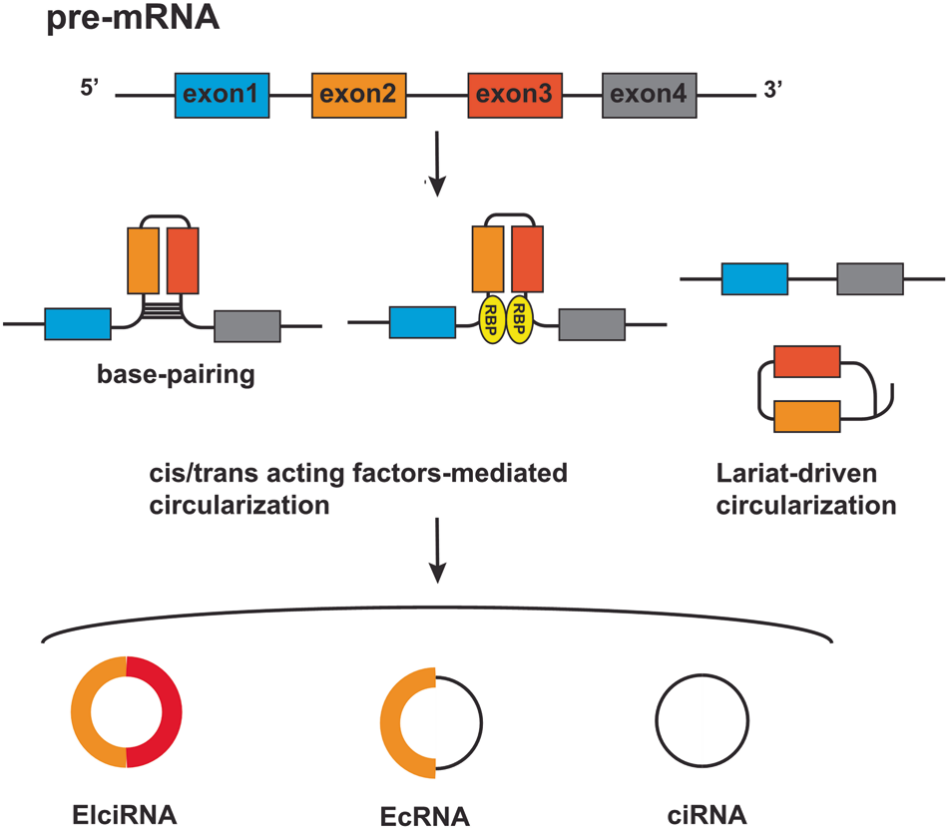
circRNA biogenesis. CircRNAs are mainly generated from the back-splicing of pre-mRNA. Cis/trans-acting factor-mediated circularization (left). As cis-acting elements, some intronic complementary sequences facilitate circularization via the formation of an intramolecular hairpin structure, by base-pairing to close the distance between the 5′ and 3′ splice sites. Meanwhile, as trans-acting factors, some RNA-binding proteins (RBPs) facilitate the production of circRNAs by binding to specific intronic motifs to bring the splice sites into proximity via self-dimerization. Lariat-driven circularization (right). In exon-skipping events, the skipped exons and introns are spliced out from the pre-mRNA and form a lariat structure, which is further spliced to form circRNAs. circRNAs can be mainly divided into three groups, based on their origin: exon-derived circRNA (EcRNA), exon–intron circRNA (EIciRNA), and intron-derived circRNA from pre-mRNA (ciRNA).

The biogenesis of circRNAs is complicated, but they are primarily produced from pre-mRNA back-splicing, which is characterized by direct binding of a 5’ downstream splice site (donor) to a 3’upstream splice stie (acceptor) via a phosphodiester bond (3’–5’)[36]. Based on the origin of circRNAs, they are mainly classified into 3 groups: exon-derived circRNA (EcRNA), intron-derived circRNA, including intronic circRNAs derived from pre-mRNA (ciRNA) and those derived from pre-tRNA (tricRNA), and exon–intron circRNA (EIciRNA) [12]. Various cis-/trans-acting factors is implicated in the regulation of circRNA generation (Fig. 1). Some intronic complementary sequences, including short inverted repeats (such as Alu repeats) [37] and non-repetitive sequences, can act as cis-acting elements to facilitate circRNA biogenesis. The circularization of exons can be mediated by these complementary sequences located in flanking introns, by forming an intramolecular hairpin structure to close the distance between the 5′ and 3′ splice sites. Short inverted repeats (approximately 30–40 nt) are sufficient for circularization; however, the sequences of the repeats affect circularization efficiency. Some sequences of low complexity, such as poly(A) tracts, suppress circularization [37]. Moreover, as trans-acting factors, some RNA-binding proteins (RBPs), including muscleblind [36] and Quaking (QKI) [38], facilitate circRNA production by binding to specific intronic motifs. QKI binds to theses intronic elements flanking circRNA-forming exons, then brings the splice sites into proximity via self-dimerization. Additionally, Nuclear factor 90 (NF90) and NF110, the immune factors mediating host immune responses to viral infections, also regulate the circularization of circRNA [39]. They contain double stranded (ds) RNA-binding domains and facilitate circRNA generation via binding to RNA pairs in flanking introns and stabilizing the base-pairing. Conversely, some RPBs, such as DExH-box helicase 9, suppress circRNA biogenesis via destabilizing the base-pairing of intronic elements.

Another model for circRNA biogenesis is called “Lariat-driven circularization” [12]. During exon-skipping events, a lariat structure is formed via splicing the skipped exons and introns out from the pre-mRNA, which is further spliced to remove intronic sequences, resulting in the generation of an EcRNA. Alternatively, a lariat structure may be formed during intron removal from pre-mRNAs, which is further spliced for ciRNA generation.

### The main mechanism underlying circRNA functions

circRNAs exhibit multiple functions in physiological and pathological processes. The sequence, secondary structure, post-transcriptional modifications and cellular location of circRNAs are related to their functions.

#### microRNA (miRNA) sponge

**Fig. 2:**
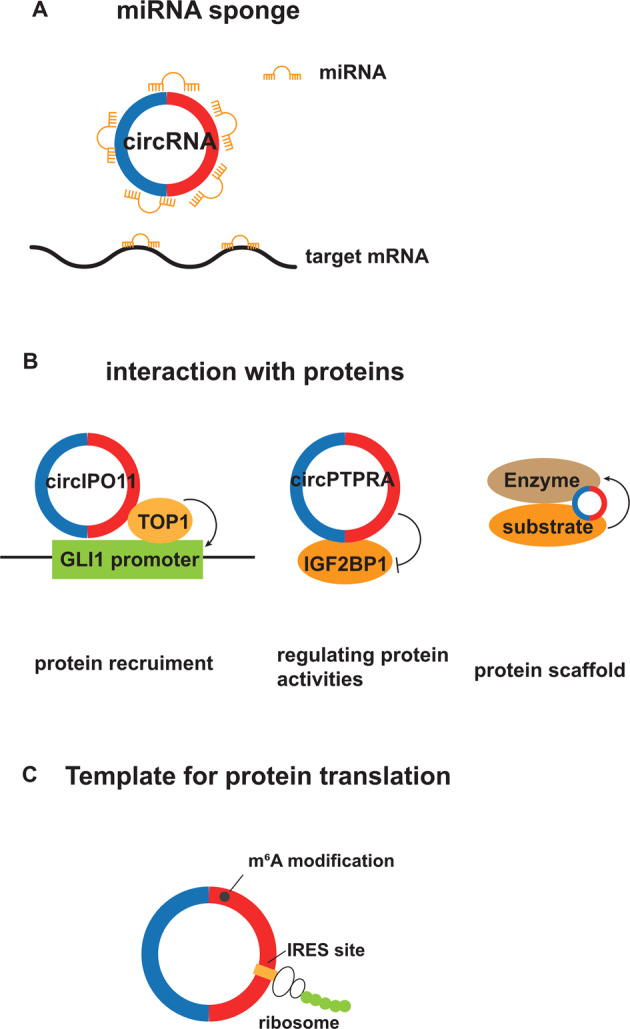
The main mechanisms underlying the functions of circRNA. **A** miRNA sponge. Some circRNAs negatively regulate miRNA-mediated gene silencing by competitively binding to miRNA and releasing its target mRNA. **B** Interaction with proteins. CircIPO11 interacts with topoisomerase 1 (TOP1) and then recruits it to the GLI1 promoter to trigger protein expression. circPTPRA interacts with IGF2BP1 and inhibits its function as an m^6^A reader. As protein scaffolds, some circRNAs interact with an enzyme and its substrate and then facilitate the association between them. **C** As a template for protein translation. Some circRNAs containing internal ribosome entry sites (IRES) and those with N6-methyladenosine (m6A) modification can serve as templates for protein translation.

The most well-studied function of circRNAs is known as miRNA sponging. Some circRNAs negatively regulate miRNA-mediated gene silencing by acting as competitive endogenous RNAs (ceRNAs) (Fig. 2A). circHERC4 was recently found to exhibit oncogenic effects in CRC, by binding to and inactivating the tumor suppressor, miR-556-5p [40]. circDOCK1 also acts as a miRNA sponge and promotes tumorigenesis in osteogenic sarcoma, via the circDOCK1/ miR-339-3p/IGF1R axis [41]. Those circRNAs with function as miRNA sponges harbor miRNA binding sites (6-, 7-, 8-mer), complementary to miRNA seed sequences. However, the miRNA binding sites in most circRNAs are very few, and the expression level of circRNAs is relatively low compared with their corresponding miRNAs. These findings mean that the ceRNA hypothesis remains controversial [42].

#### Interactions with proteins

Some circRNAs bind to proteins and recruit them to certain subcellular compartments (Fig. 2B) [26, 43, 44]. Gu and colleagues described a circRNA, circIPO11, that is required to maintain the self-renewal of cancer-initiating cells in HCC [26]. circIPO11 was shown to trigger the expression of GLI family zinc finger protein1 via recruiting topoisomerase 1 to its promoter, and result in the activation of Hedgehog signaling. circMYH9, an intron-derived circRNA, was found to recruit hnRNPA2B1 in the nucleus and bound to p53 pre-mRNA to maintain its stability. In this way, circMYH9 promotes CRC cell proliferation in a p53-dependent manner [44]. Some circRNAs interact with proteins and suppress their activities [45–48]. circPTPRA was shown to suppress the progression of bladder cancer, through its interaction with IGF2BP1, an N6-methyladenosine (m6A) reader, and blocking the IGF2BP1-mediated recognition of m6A-modified RNAs [47]. Furthermore, some circRNAs function as protein scaffolds to affect interactions among proteins [49, 50]. As an example, circRNA-DOPEY2 facilitates the association between the E3 ligase TRIM25 and its substrate cytoplasmic polyadenylation element binding protein (CPEB4), leading to increased CPEB4 degradation [50]. As a result, the circRNA promotes chemosensitivity in esophageal cancer cells.

#### Translation

Although circRNAs were previously identified as non-coding RNAs, because they lacked a 5′ cap structure and a 3′ poly-A tail, recently some circRNAs containing internal ribosome entry sites (IRES) have been revealed to serves as templates for protein synthesis (Fig. 2C). We recently described AXIN1–295aa as a novel oncogenic factor in GC, which is encoded by circAXIN1with an IRES element [16]. We determined that this protein promotes GC development by interacting with APC and promoting the activation of Wnt/β-catenin signaling. Several circRNAs with m6A modification also exhibit protein-coding potential. Li et al. identified a novel oncogenic circRNA, circARHGAP35, which harbors an open reading frame (ORF) with an m6A-modified start codon and encodes a protein, referred to as circARHGAP35 protein. They further determined that the circRNA-derived protein shows antithetical expression and function compared with its linear partner and enhances the development of HCC and CRC via the interaction with TFII-I protein [51]. Furthermore, circDIDO1 with IRES, ORF, and m6A modification encodes a novel tumor suppressor protein, which suppresses GC progression by interacting with peroxiredoxin 2 (PRDX2) and poly ADP-ribose polymerase 1 (PARP1), affecting the activity or stability of these proteins [52].

## Autophagy

circRNAs have both stimulatory and inhibitory effects on autophagy, and they mainly regulate the process of autophagy via ceRNA mechanisms. circRNA-mediated autophagy is implicated in cancer development and plays vital roles in cell proliferation, metastasis, chemoresistance, and apoptosis.

### The roles played by circRNAs in autophagic processes

**Fig. 3:**
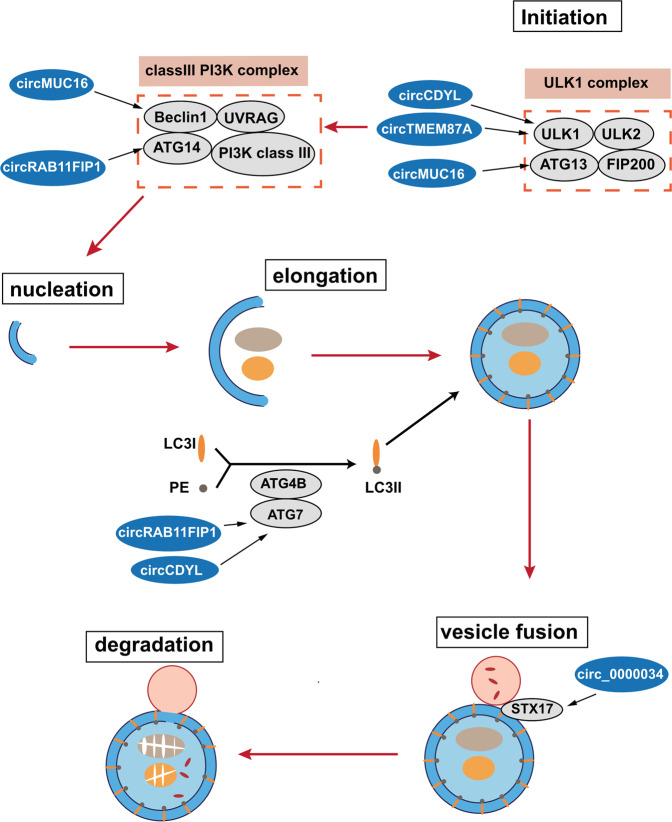
The role of circRNAs in the process of autophagy. The autophagic processes can be divided into five stages: initiation, autophagosome nucleation, the elongation of the autophagosome membrane, the fusion of the autophagosome with a lysosome, and the degradation of the autophagic cargo. circRNAs are involved in these processes by interacting with autophagy-related proteins or regulating their expression.

There are mainly five stages in the autophagic processes: initiation, autophagosome nucleation, autophagosome membrane elongation, the fusion between the autophagosome and a lysosome, and the degradation of the autophagic cargo [31] (Fig. 3). circRNAs are implicated in these processes via interacting with autophagy-related proteins or regulating their expression. During the initiation stage, a unc-51-like kinase 1 (ULK1) complex, composed of ULK1, ULK2, autophagy-related 13 (ATG13), and FIP200 is activated [31]. Several circRNAs are associated with the initiation stage. circCDYL and circTMEM87A regulate the expression of ULK1 via acting as the sponges of miR-1275 and miR-142-5p, respectively, and further promote autophagy [53, 54]. circMUC16 promotes ATG13 expression and facilitates autophagy via directly binding to ATG13 in epithelial ovarian cancer (EOC) [55]. Then, the ULK1 complex induces the activation of a class III PI3K complex (containing Beclin1, ATG14, UVRAG, and class III PI3K), which mediates autophagosome nucleation. During this process, circMUC16 enhances autophagy via the circMUC16/ miR-199a-5p/Beclin1 axis [55]. circRAB11FIP1 regulates ATG14 expression by sponging miR-129 [56]. The complex of ATG5-ATG12 mediates the elongation of the autophagosome membrane, via conjugating with ATG16 protein. Meanwhile, LC3-II is formed via the conjugation of LC3-I with lipid phosphatidylethanolamine (PE), followed by being recruited to the membrane of autophagosome. This process is mediated by the ATG4B-ATG7 complex. circRAB11FIP1 and circCDYL are reported to regulate ATG7 expression via serving as the sponges of miR-129 and miR-1275, respectively, and promote autophagy [53, 56]. Subsequently, the fusion between autophagosomes and lysosomes is facilitated by the SNARE protein syntaxin 17 (STX17), resulting in the degradation of autophagic cargoes. Circ_0000034 is implicated in the process by regulating the miR-361-3p/STX17 axis [57].

### The multiple signaling pathways associated with circRNA-mediated regulation of autophagy

**Fig. 4:**
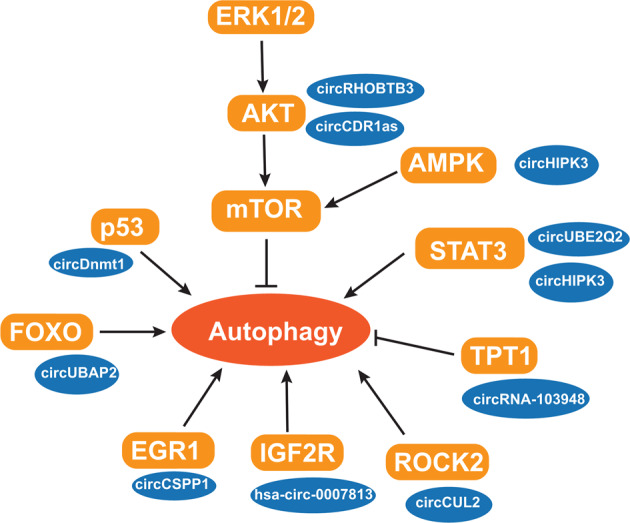
Multiple signaling pathways are implicated in the regulation of autophagy by circRNAs. circRNAs participate in multiple signaling pathways to regulate autophagy, by acting as miRNA sponges or through interactions with proteins.

circRNAs regulate autophagy via a diverse range of signaling pathways, as described below (Fig. 4).

#### Signal transducer and activator of transcription 3 (STAT3)

STAT3 signaling, a stress response pathway, has been reported to participate in regulating autophagy [58]. STAT3 proteins located in various subcellular compartments can affect the autophagic process in diverse ways [58]. Phosphorylated STAT3 proteins form dimers and enter the nucleus to regulate the expression of autophagy-associated genes, including Bcl-2, BECN1, and PIK3C3. Cytoplasmic unphosphorylated STAT3 suppresses autophagy by sequestering FOXO1, FOXO3, and EIF2AK2. circUBE2Q2 was shown to suppress miR-370-3p/STAT3-mediated autophagy and promote GC development. Its knockdown was found to significantly decrease STAT3, p-STAT3, and Bcl-2 levels, resulting in increased autophagy [35]. Chen et al. also identified a circRNA, circHIPK3, as a key autophagy regulator in lung cancer [59]. circHIPK3 promotes cancer progression and suppresses autophagy, partially by sponging miR-124-3p and regulating the expression of its target, STAT3.

#### Mammalian target of rapamycin (mTOR)

mTOR is a key regulator of autophagy. As a serine–threonine kinase, mTOR forms an mTORC1 complex with mLST8 and RAPTOR proteins, and inhibits the ULK1 complex by inducing the phosphorylation of the complex components, including ULK1/2 and ATG13 [60]. Several signaling pathways participate in autophagy via the regulation of mTOR activity, including the adenosine monophosphate-activated protein kinase (AMPK), phosphoinositide 3-kinase (PI3K)/protein kinase (AKT) and extracellular-signal-regulated kinase (ERK) signaling pathways [61]. Yang et al. identified a pro-autophagic circRNA, circRHOBTB3, in PDAC [34]. This circRNA functions as a miRNA sponge, and relieves miR-600-mediated inhibition of expression of NACC1, and then facilitates autophagy via suppresses the Akt/mTOR signaling. circCDR1as is reported to promote autophagy and the development of oral squamous cell carcinoma (OSCC), by sponging miR-671-5p [62]. It enhances hypoxia-induced autophagy via the upregulation of the AKT and ERK1/2 pathways and inhibiting mTOR activity. Furthermore, circPAN3 was determined to facilitate autophagy and chemoresistance in acute myeloid leukemia through AMPK-mTOR signaling [63].

#### p53

The p53 pathway exhibits both promoting and inhibiting effects on autophagy, according to its cellular localization [64–66]. Nuclear p53 promotes autophagy via transactivating the expression of pro-autophagic proteins, such as Sestrin1 and Sestrin2 [67], AMPK [68, 69] and damage-regulated autophagy modulator (DRAM) [70, 71], while cytoplasmic p53 is involved in autophagy inhibition. circDnmt1 induces autophagy and increases the survival capacity of cells in breast cancer (BCa), by promoting the transport of p53 and AUF1 into cellular nucleus [15]. Nuclear p53 stimulates autophagy, while nuclear AUF1 increases the expression of Dnmt1, which inhibits p53 transcription.

#### Forkhead box class O (FOXO)

The effects of FOXO transcription factors on autophagy are also dependent on their cellular location. The cytoplasmic FOXOs interact with autophagy-associated proteins directly, while the nuclear ones regulate autophagy-related gene expression [72, 73]. Chen and colleagues demonstrated the pro-autophagic and oncogenic effects of circUBAP2 in CRC, which regulates the expression of miR-582-5p-targeted FOXO1 by sponging this circRNA [74]. circMRPS35 induces autophagy in osteosarcoma via interacting with KAT6B, and further promoting FOXO3 expression [75].

#### Other pathways associated with circRNA-mediated autophagy

Early growth response factor 1 (EGR1) was identified as a transcriptional regulator of autophagy, modulating the expression of autophagy-related genes [76]. CircCSPP1 facilitates autophagy and enhances the progression of prostate cancer (PCa), via the miR-520h/EGR1 axis [77]. Insulin-like growth factor 2 receptor (IGF2R) also regulates the autophagic processes. The loss of IGF2R results in lysosome dysfunction and autophagy inhibition [78]. hsa_circ_0007813 was shown to exhibit pro-autophagic and oncogenic effects on the development of bladder cancer, by modulating the expression of hsa-miR-361-3p-targeted IGF2R [79]. ROCK2 is also implicated in autophagy [80]. circCUL2 activates ROCK2-mediated autophagy via sponging miR-142-3p and then modulates chemoresistance in GC [81]. Furthermore, the tRNA splicing enzyme TPT1 has been reported to negatively regulate autophagy [82]. circRNA_103948 was found to inhibit autophagy in CRC by regulating the expression of miR-1236-3p-targeted TPT1 [83].

### The diverse effects of circRNA-mediated autophagy in cancer

circRNA-mediated autophagy plays vital roles in multiple aspects of tumor progression, including cellular proliferation, invasion and metastasis. It is also associated with cancer escape mechanisms and renders cancer cells resistant to chemotherapy.

#### The role of circRNA-mediated autophagy in tumor development

circRNA-mediated autophagy can exhibit either promoting or inhibiting effects on cancer development across different tumor types (Table 1). In BCa, autophagy enhanced by circDnmt1 and circCDYL promotes the malignant progression of tumors [15, 53], while hsa_circ_0000515 knockdown induces autophagy and apoptosis but suppresses cellular proliferation and invasion in cervical cancer, suggesting an anti-tumor effect of autophagy [84]. circRHOBTB3-mediated autophagy was found to promote cell proliferation in PDAC [34]. It has even been noted that autophagy can have antithetical effects on cancer progression, even within the same tumor type. The silencing of circUBE2Q2 increased autophagy but suppressed glycolysis, cell proliferation, invasion, and migration in GC [35]. Meanwhile, circCUL2 was found to activate autophagy but inhibit tumorigenicity in GC [81]. These findings suggest that autophagy regulated by circUBE2Q2 and circCUL2 exhibits anti-tumor activity in GC, whereas circTMEM87A-mediated autophagy positively regulates GC progression [54]. Similarly, circUBAP2 promotes autophagic processes and enhances the development and metastasis of CRC [74], while circRNA_103948 suppresses autophagy and promotes the progression of CRC [83]. These findings indicate the opposing effects of autophagy on CRC progression. Therefore, the effects of autophagy on tumorigenesis and progression may be dependent on tumor types, the genetic context of cells, and specific types of cellular stresses. An understanding of the mechanisms underlying how circRNAs regulate autophagic processes and how autophagy affects tumorigenesis is thus important for the development of circRNA-based cancer therapy strategies.

**Table 1:** The roles of autophagy-associated circRNAs in cancer development.

circRNA	Roles in autophagy	Mechanism	Target	Cancer types	Roles in cancer development	Dysregulation in cancer(up/down)	References
circDnmt1	Promote	Recruitment of proteins	P53 and AUF1	Bca	Oncogene	Up	[15]
circCSPP1	Promote	ceRNA	miR-520h/EGR1	PCa	Oncogene	Up	[77]
circUBAP2	Promote	ceRNA	miR-582-5p/FOXO1	CRC	Oncogene	Up	[74]
circRHOBTB3	Promote	ceRNA	miR-600/NACC1	PDAC	Oncogene	Up	[34]
circRAB11FIP1	Promote	ceRNA	miR-129/ATG7 and ATG14	EOC	Oncogene	Up	[56]
circCDYL	Promote	ceRNA	miR-1275/ATG7 and ULK1	BCa	Oncogene	Up	[53]
circMUC16	Promote	ceRNA; Interaction with protein	miR-199a-5p/Beclin1 and RUNX1; ATG13	EOC	Oncogene	Up	[55]
hsa_circ_0007813	Promote	ceRNA	hsa-miR-361-3p/IGF2R	bladder cancer	Oncogene	Up	[79]
circTMEM87A	Promote	ceRNA	miR-142-5p/ULK1	GC	Oncogene	Up	[54]
circCDR1as	Promote	ceRNA	miR-671-5p	OSCC	Oncogene	Up	[62]
circ_0000034	Promote	ceRNA	miR-361-3p/STX17	RB	Oncogene	Up	[57]
circMRPS35	Promote	Interaction with protein	KAT6B/FOXO3	osteosarcoma	Anti-oncogene	_	[75]
circUBE2Q2	Suppress	ceRNA	miR-370-3p/STAT3	GC	Oncogene	Up	[35]
circHIPK3	Suppress	ceRNA	miR-124-3p/STAT3	NSCLC	Oncogene	_	[59]
hsa_circ_0000515	Suppress	ceRNA	miR-326/ELK1	Cervical cancer	Oncogene	Up	[84]
circRNA_103948	Suppress	ceRNA	miR-1236-3p/TPT1	CRC	Oncogene	Up	[83]

*Bca* breast cancer, *PCa* prostate cancer, *GC* gastric cancer, CRC colorectal cancer, *PDAC* pancreatic ductal adenocarcinoma, *EOC* epithelial ovarian cancer, *NSCLC* non-small cell lung cancer, *OSCC* oral squamous cell carcinoma, *RB* retinoblastoma.

#### The role of circRNA-mediated autophagy in chemoresistance of cancer cells

circRNAs are also implicated in the chemoresistance of tumor cells by regulating autophagy (Table 2). Cisplatin, a platinum-based reagent, has been widely used in therapy for solid cancers, such as ovarian, lung, colorectal, bladder, and head and neck cancers [85, 86]. However, chemoresistance often develops following cisplatin-based therapy, limiting its clinical utility [87]. circPARD3 facilitates chemoresistance of cancer cells to cisplatin in laryngeal squamous cell carcinoma via regulating PRKCI-AKT-mTOR signaling and then suppressing autophagy [88]. The silencing of circPARD3 increases the sensitivity of cancer cells to this drug. circCUL2 also affects the resistance of GC cells to cisplatin via modulating miR-142-3p/ROCK2-mediated autophagy [81]. Zhong and colleagues described a circRNA, circRNA_100565, associated with the resistance of non-small cell lung cancer (NSCLC) cells to cisplatin [89]. The upregulation of circRNA_100565 was observed in cisplatin-resistant NSCLC cell lines and tissues. Furtherly, this circRNA promotes autophagy and cell proliferation, but suppresses apoptosis via the miR-337-3p/ADAM metallopeptidase domain 28 (ADAM28) axis, resulting in the enhancement of cisplatin resistance. In addition, several circRNAs contribute to the resistance of cancer cells to apatinib, a selective inhibitor of vascular endothelial growth factor receptor 2 (VEGFR2), which also exhibits anti-tumor activity in solid tumors [90]. Ma et al. showed that circRACGAP1 is involved in the resistance of cancer cells to Apatinib in GC via regulating the miR-3657/ATG7-mediated autophagy. circRACGAP1 knockdown sensitized GC cells to the drug via suppressing autophagy [91]. Additionally, this circRNA regulates gefitinib resistance in NSCLC via targeting miR-144-5p/CDKL1. circRACGAP1 knockdown significantly increases the gefitinib sensitivity in NSCLC cells [92]. The upregulation of circ_0009910 was noted in the serum of imatinib-resistant patients with chronic myeloid leukemia (CML) [93]. circ_0009910 enhances the resistance of CML cells to imatinib via sponging miR-34a-5p and then regulating ULK1-mediated autophagy. Thus, the roles played by circRNAs in the chemoresistance of cancer cells suggest that targeting circRNA-mediated autophagy is a potential strategy to attenuate chemoresistance in patients with advanced cancer.

**Table 2:** The roles of autophagy-associated circRNAs in chemoresistance.

circRNA	Roles in autophagy	Mechanism	Target	Anti-cancer reagents	Cancer types	Roles in cancer development	Dysregulation in cancer(up/down)	References
circCUL2	Promote	ceRNA	miR-142-3p/ROCK2	Cisplatin	GC	Anti-oncogene	Down	[81]
circRNA_100565	Promote	ceRNA	miR-337-3p/ADAM28	Cisplatin	NSCLC	Oncogene	Up	[89]
circRACGAP1	Promote	ceRNA	miR-3657/ATG7	Apatinib	GC	Oncogene	Up	[91]
circRACGAP1	_	ceRNA	miR-144-5p/CDKL1	Gefitinib	NSCLC	Oncogene	Up	[92]
circ_0009910	Promote	ceRNA	miR-34a-5p/ULK1	Imatinib	CML	Oncogene	Up	[93]
circCPM	Promote	ceRNA	miR-21-3p/PRKAA2	5-FU	GC	Oncogene	Up	[103]
circ_0035483	Promote	ceRNA	miR-335/cyclinB1	Gemcitabine	RCC	Oncogene	Up	[104]
circPARD3	Suppress	ceRNA	miR-145-5p/PRKC1	Cisplatin	LSCC	Oncogene	Up	[88]

*LSCC* laryngeal squamous cell carcinoma, *GC* gastric cancer, *NSCLC* non-small cell lung cancer, *CML* chronic myeloid leukemia, *RCC* renal clear cell carcinoma.

## The potential of autophagy-associated circRNAs in cancer treatment

With the increasing understanding of the regulatory roles played by circRNAs in autophagy and cancer development, the potential of circRNAs in tumor therapy is attracting more and more attention. The expression patterns of circRNAs have been shown that be associated with the clinicopathological characteristics of cancer. circHIPK3 plays an important role in regulating autophagy in NSCLC [59]. circHIPK3 and its linear partner (linHIPK3) exert the opposite regulatory effects on autophagy.The ratio between circHIPK3 and linHIPK3 (the C:L ratio) reflects the level of autophagy, with a low C:L ratio inducing autophagy in NSCLC. Additionally, a high C:L ratio indicates poor survival in patients with advanced-stage NSCLC, therefore it was suggested that the ratio can be used as a prognostic factor in NSCLC. Moreover, circMUC16 was reported to promote autophagy in EOC [55]. circMUC16 is upregulated in EOC tissues and the expression level of this circRNA is closely related to EOC development, both in terms of stage and grade. Therefore, circRNAs exhibit potential as novel targets for cancer diagnosis and therapy.

Some circRNAs are detectable in body fluids, such as peripheral blood, saliva, and urine, providing the possibility that they could be used as non-invasive diagnostic and prognostic biomarkers for numerous human diseases, including cancers [94]. Thus, the circRNAs enriched in exosomes have received more and more attention. Exosomes are tiny membrane vesicles circulating in body fluids, which are secreted by various types of cells, such as cancer cells [95]. They contain various substances, including circRNAs, other nucleic acids, lipids, and proteins, and are associated with intercellular communication and the formation of the tumor microenvironment and premetastatic niches [94, 96, 97]. Exosomes carry circRNAs to target cells and, at the same time, increase their stability [94]. Increasing numbers of cancer-derived exosomal circRNAs have been identified, and their expression level is related to tumor progression. As they are abundant, stable, easy to detect, and show specific expression patterns in cancer, these exosomal circRNAs are considered as novel potential biomarkers of cancer diagnosis and prognosis. Rao et al. compared circRNA levels in plasma exosomes between GC patients and healthy donors to identify the expression profile of exosomal circRNAs in GC [98]. They found 620 upregulated and 440 downregulated exosomal circRNAs, which may participate in GC development. Additionally, 1195 and 1147 dysregulated exosome-derived circRNAs were identified in localized and metastatic BCa, respectively [99]. Compared with patients with localized BCa, 480 exosome-derived circRNAs were dysregulated in metastatic BCa patients, indicating their role in BCa metastasis. Moreover, circSHKBP1 is upregulated in cancer tissues and serum of GC patients [100]. Its increased expression is linked to poor survival and advanced TNM stage. The exosomal circSHKBP1 enhances GC development via the miR-582-3p/HUR/VEGF axis and its level significantly decreases following gastrectomy. Therefore, exosomal circSHKBP1 might be a potential biomarker for GC. The enrichment of circSATB2 was found in serum exosomes from NSCLC patients and is linked to metastasis [101]. This exosomal circRNA, which can be detected with high sensitivity and specificity, also exhibit potential as a diagnostic biomarker for NSCLC. The expression patterns of autophagy-associated circRNAs in cancer-derived exosomes and their correlation with clinicopathological characteristics of cancers should be investigated further. The regulatory roles and the molecular mechanisms of these exosomal circRNAs during tumor development also need to be further elucidated.

Chemotherapy is primarily used for the treatment of metastatic cancers. However, that multidrug resistance is usually developed in patients renders cancer treatment more difficult. Autophagy-associated circRNAs play vital roles in chemoresistance development; therefore, these circRNAs can be targeted to enhance the sensitivity of cancer cells to anti-cancer reagents. The drug 5-fluorouracil (5-FU) is frequently used for the treatment of advanced GC [102]. Fang et al. reported that circCPM is upregulated in GC cell lines and tissues with resistance to 5-FU [103]. circCPM promotes PRKAA2-mediated autophagy by sponging miR-21-3p and then enhances 5-FU resistance of GC cells. Therefore, targeting circCPM may represent a novel therapeutic strategy to reverse 5-FU resistance in GC. Hsa_circ_0035483 promotes gemcitabine-induced autophagy and enhances gemcitabine resistance in renal clear cell carcinoma (RCC) via sponging has_miR_335 [104]. circ_0035483 knockdown enhances the gemcitabine sensitivity, suggesting its potential as a novel target of treatment to reverse chemoresistance in RCC.

## Conclusion

circRNAs is implicated in the regulation of autophagy, via multiple signaling pathways. circRNA-mediated autophagy plays vital roles in multiple aspects of tumor progression, and exhibits both pro- and anti-tumor effects, in a context-dependent manner. Therefore, an understanding of the mechanisms by which circRNA-mediated autophagy affects tumorigenesis and cancer progression is particularly important for the development of novel circRNA-based cancer therapeutic strategies. Furtherly, circRNAs also participate in the chemoresistance of cancer cells through the regulation of autophagy. The combination of traditional chemotherapy and new therapeutic strategies targeting autophagy-associated circRNAs may lead to more effective treatments for cancer. Additionally, some circRNAs enriched in exosomes are stable and detectable in body fluids, while the expression levels of circRNAs are linked to clinicopathological characteristics of cancers. Therefore, circRNAs are also considered to represent novel and potentially useful diagnostic and prognostic biomarkers of cancer.

## Data Availability

All the data used to support the findings of this study are available in the paper.
